# Urachal Carcinomas: A Comprehensive Systematic Review and Meta-analysis

**DOI:** 10.1590/S1677-5538.IBJU.2024.0665

**Published:** 2025-01-21

**Authors:** Caio Vinícius Suartz, Lucas Motta Martinez, Marcelo Henrique Lima Silvestre, Richard Dobrucki de Lima, Pedro Henrique Souza Brito, Ketlyn Assunção Galhardo, Roberto Iglesias Lopes, Victor Hondo Silva de Moraes, Caio Mazzonetto Teofilo de Moraes, Luana Covatti, Maria Fernanda Dias Azevedo, Lucas Schenk de Almeida, Debora Narumi Demitrol Setoue, Natália Doratioto Serrano Faria Braz, José Bessa, Fernando Korkes, Leonardo O. Reis, Kátia Ramos Moreira Leite, William Carlos Nahas, Paul Toren, Leopoldo Alves Ribeiro

**Affiliations:** 1 Northern Ontario School of Medicine Department of Urology Thunder Bay Canada Department of Urology, Northern Ontario School of Medicine, Thunder Bay, Ontario, Canada; 2 CHU de Québec-Université Laval Quebec City Canada CHU de Québec-Université Laval, Quebec City, Quebec, Canada; 3 Universidade de São Paulo Instituto do Câncer de São Paulo Divisão de Urologia São Paulo SP Brasil Divisão de Urologia, Instituto do Câncer de São Paulo, Universidade de São Paulo - USP, São Paulo, SP, Brasil; 4 Hospital Israelita Albert Einstein Faculdade Israelita Albert Einstein de Ciências da Saúde São Paulo Brasil Faculdade Israelita Albert Einstein de Ciências da Saúde (FICSAE), Hospital Israelita Albert Einstein (HIAE), São Paulo, Brasil; 5 Universidade Estadual de Feira de Santana Feira de Santana BA Brasil Universidade Estadual de Feira de Santana - UEFS, Secretaria de Saúde, Feira de Santana, BA, Brasil; 6 Faculdade de Medicina do ABC Divisão de Urologia, Oncologia Urológica Santo André SP Brasil Divisão de Urologia, Oncologia Urológica, Faculdade de Medicina do ABC - FMABC, Santo André, SP, Brasil; 7 Universidade Estadual de Campinas Divisão de UroScience São Paulo Brasil Divisão de Oncologia Urológica, Divisão de UroScience, Universidade Estadual de Campinas - Unicamp, Campinas, São Paulo, Brasil; 8 Pontifícia Universidade Católica de Campinas Divisão de ImunOncologia São Paulo Brasil Divisão de ImunOncologia, Pontifícia Universidade Católica de Campinas, Campinas, São Paulo, Brasil

**Keywords:** Urachal cancer [Supplementary Concept], Cystectomy, Systematic Review [Publication Type]

## Abstract

**Objective:**

This systematic review and meta-analysis aim to consolidate current evidence on the diagnosis, epidemiology, and treatment of urachal carcinoma, a rare malignancy with limited data.

**Materials and Methods:**

A systematic search of PubMed/MEDLINE was conducted up to September 2024 to identify studies involving patients with urachal carcinoma, reporting clinical epidemiological characteristics, diagnostic strategies, histopathological findings, tumor staging, treatment modalities, and oncological outcomes. Extracted data were systematically synthesized, and statistical analyses, including a single-arm meta-analysis, were performed to comprehensively evaluate oncological outcomes.

**Results:**

Our study includes 1,901 cases of urachal carcinoma from 50 studies. The findings support the oncologic advantage of *en-bloc* resection with umbilectomy in localized disease, demonstrating improved survival outcomes and reduced recurrence rates. In the adjuvant setting, those receiving cisplatin-based therapy presented the best response, with 65.73% with no disease progression; similarly, in the metastatic disease, cisplatin-based regimens seem to have better responses in metastatic disease. The single-arm meta-analysis estimated a 5-year overall survival rate of 51% (95% CI: 0.49–0.54). Tumor recurrence was documented in 35% of cases (95% CI: 0.25–0.45), with local recurrence occurring in 28% (95% CI: 0.18–0.38), with the average time to recurrence of 27.6 months.

**Conclusion:**

Our study provides the most comprehensive review of urachal carcinoma to date, providing evidence to guide clinical decisions. It underscores the oncologic benefits of *en-bloc* resection with umbilectomy and specific chemotherapeutic regimens. Emerging alternative therapies also show potential, highlighting the need for further research to optimize patient outcomes.

## INTRODUCTION

The urachus, a remnant of the embryonic allantois, typically becomes nonfunctional after birth. This structure forms during early development as the allantois regresses into a tubular connection between the urinary bladder and the umbilicus. By the end of gestation, it generally transforms into a fibrous cord that fuses with the obliterated umbilical arteries, creating the median umbilical ligament (1). However, a residual urachal structure remains in around one-third of adults, often presenting as a tubular or cystic formation lined by epithelium. This developmental remnant may serve as a site for urachal carcinoma (UrC) (1, 2).

UrC differs in pathological and clinical features from bladder carcinomas, highlighting their distinct origins and characteristics. It is rare, comprising less than 1% of all bladder cancer cases. Incidence estimates range from 0.022 to 0.060 per 100,000 person-years (3).

The clinical-epidemiological characteristics, surgical and clinical management, and oncological outcomes of UrC are predominantly supported by weak evidence derived from case reports, small case series, or population-based databases with incomplete information and significant missing data. When analyzed in isolation, these limitations make it challenging to apply the findings effectively to clinical practice, given the rarity of the disease.

Current controversies in the literature include the necessity of umbilical resection in conjunction with cystectomy, the indication and extent of lymphadenectomy, as well as diagnostic and prognostic criteria, all of which remain subjects of debate with conflicting results. This systematic review and meta-analysis aim to synthesize the available literature to provide more robust scientific evidence, facilitating evidence-based management for this rare malignancy.

## MATERIALS AND METHODS

### Literature search

The study was conducted in strict compliance with the Preferred Reporting Items for Systematic Reviews and Meta-Analysis (PRISMA) (4) statement and registered in the PROSPERO international database of prospectively registered systematic reviews (CRD42024562424).

Based on the Patient-Intervention-comparator-outcome-study design (PICOS) criteria (5), a research question was established: What is the current evidence regarding the clinical, epidemiological characteristics, management strategies, and oncological outcomes of urachal carcinoma?

The search strategy was (urachal carcinoma) OR (urachal adenocarcinoma) OR (urachal cancer), and we searched in PubMed/MEDLINE up to September 2024. We also checked the bibliographies of the included studies for further references to relevant trials. We included all case series, cohort studies, and randomized trials, all involving patients over 18 years of age with UrC without language restrictions. We excluded governmental databases, case reports, case series with fewer than eight patients or with incomplete information, editorial letters, expert opinions, and literature reviews.

Two independent authors screened all retrieved records. Discrepancies were resolved by discussion with a third review. If relevant to the present review, the full text of the screened papers was selected.

### Data extraction and endpoints

All variables were entered into a spreadsheet for analysis, and another author made cross-validation. The mean and standard deviation for continuous variables were recorded from the included studies. For variables reported as median and interquartile range, the original data were converted to mean and standard deviation (6).

The variables extracted included study design, patient gender, age, comorbidities, clinical symptoms, urinary cytology results, cystoscopy utilization, preoperative oncological markers, imaging modalities employed, histological classification, clinical and pathological staging, surgical approach type, lymphadenectomy and its template, performance of umbilectomy, administration of chemotherapy for neoadjuvant, adjuvant or salvage treatment and oncological outcomes.

## RESULTS

### Literature screening

**Figure 1:**
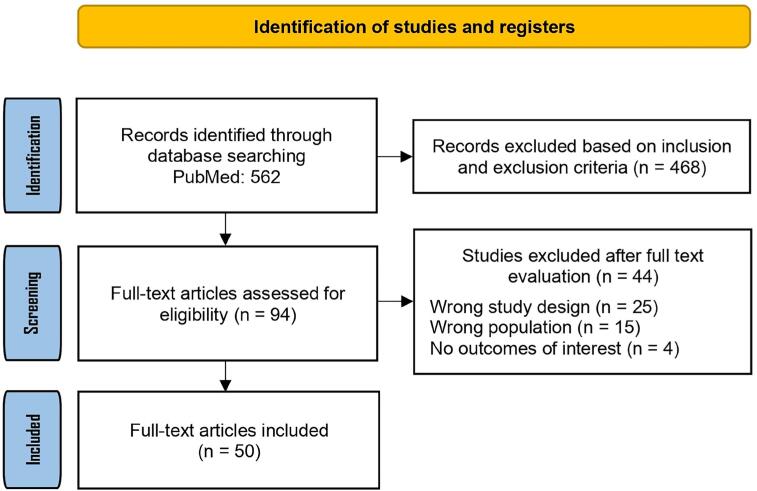
PRISMA flow chart of the selected articles.

The literature search retrieved 562 records, which were screened by title and abstract. Of these, 468 were excluded because they were irrelevant to the study's aim. We then reviewed the full texts of the remaining 94 studies to assess their eligibility. A total of 44 studies were excluded due to inappropriate study design, leaving 50 studies for inclusion in the final analysis (7–57), shown in the supplementary Table-1. Figure-1 presents the PRISMA flowchart summarizing the literature search and selection process.

### Study characteristics

All included studies were retrospective and comprised a total of 1,901 patients. The United States had the highest number of patients in the case series (N=715; 37.61%), followed by China (N=364; 19.15%) and South Korea (N=318; 16.73%). All clinical and epidemiological characteristics of the studies are summarized in Table-1.

**Table 1:** Clinical, epidemiological, and pathological characteristics of the included studies.

Characteristic; n (%)		Overall population
Number of patients		1,901
Age at primary treatment (Years); Mean (SD)		51 (±3.31)
**Gender**		1794
	Male	1,148 (63.99)
	Female	646 (36.01)
Smoker		200 (10.52)
**Clinical signs and symptoms**		1210
	Hematuria	865 (45.50)
	Abdominal pain	122 (6.41)
	Palpable mass	95 (4.99)
	Mucouria	52 (2.73)
	Dysuria	36 (1.89)
	Lower urinary tract symptoms	35 (1.84)
	Omphalorrhoea	5 (0.26)
**Diagnostic performance; Mean sensitivity (SD)**		
	Urinary cytology	0.22 (0.19)
	Cystoscopy	0.79 (0.19)
Calcification at tomography; Prevalence (%)		35.54
**Imaging exams**		952
	Computed Tomography Scan	665 (69.85)
	18F-Fluorodeoxyglucose Positron Emission Tomography	133 (13.97)
	Ultrasonography	92 (9.6)
	Laparoscopy	35 (3.67)
	Magnetic Resonance Imaging	27 (2.83)
**Oncologic marker, n**° **patients positive/n**° **patients tested (sensitivity)**		
	CEA	404 / 499 (0.80)
	CA19-9	403 / 476 (0.84)
	CA125	20 / 66 (0.30)
	Alpha fetoprotein	1/15 (0.06)
	CA 15-3	3 / 50 (0.06)
**Histology**		1264 (100)
Mucinous		508 (40.19)
	Enteric / Intestinal	426 (33.70)
	Mixed	74 (5.85)
	Signet Ring Cells	67 (5.30)
	Urothelial	70 (5.54)
	Squamous cells	11 (0.87)
	Neuroendocrine	1 (0.08)
	Not specified	107 (8.47)
**Sheldon Classification**		1,107 (100)
	I	21 (1.90)
	II	78 (7.05)
	IIIA	262 (23.67)
	IIIB	487 (43.99)
	IIIC	62 (5.60)
	IIID	27 (2.44)
	IVA	94 (8.49)
	IVB	76 (6.87)
**Mayo Classification**		814 (100)
	I	179 (21.99)
	II	385 (47.30)
	III	93 (11.43)
	IV	157 (19.29)
**TNM Staging**		579 (100)
	pT0	4 (0.69)
	pT1	27 (4.66)
	pT2	148 (25.56)
	pT3	316 (54.57)
	pT4	84 (14.50)
**Number of patients with metastasis at diagnosis**		297 (15.62)

SD = Standard deviation

### Epidemiology and Clinical Characteristics

The mean age at primary treatment was 51 years (±3.31), with a predominance of male patients 64%. Regarding most frequent comorbidities, 24% of patients had systemic arterial hypertension, and 13.3% had diabetes mellitus. In terms of clinical presentation, macroscopic hematuria was the most common symptom (45.5%), followed by abdominal pain (6.4%) and palpable abdominal mass (5%).

### Diagnostic Methods

Concerning the prevalence of imaging modalities utilized, most patients underwent abdominal and pelvic computed tomography (CT) (69.85%). The second most performed imaging test was 18F-fluorodeoxyglucose (18F-FDG) positron emission tomography (PET)/CT (13.65%), while ultrasonography was used in only 9.6% of cases. The use of 18F-FDG PET CT has shown value in identifying metastatic sites that may be missed by other imaging methods, especially during follow-up. However, it does not appear to offer substantial additional insights over CT, which remains the preferred and most reliable tool for initial diagnosis and staging. Urinary cytology, when performed, had an average sensitivity of 22%, whereas cystoscopy demonstrated a higher average sensitivity of 79%. Calcification of the lesion on CT was previously reported in 50%–70% of patients, but in our systematic review was observed in only 35.5% of patients (58).

Regarding serum biomarkers, some studies utilized CEA, CA 19-9, CA 125, CA 15-3, and alpha-fetoprotein. The two markers with the highest sensitivity for UrC were CA 19-9 (84%) and CEA (80%).

Cystoscopy had a sensitivity of 0.79 (±0.19). In most cases where urachal carcinoma is detected, there is a protrusion in the bladder mucosa or a lesion that shows growth from the external region towards the bladder urothelium, contrasting with the typical tumor progression of urothelial carcinoma. A biopsy is an essential tool for diagnosis, especially in cases of atypical localization or advanced clinical staging. In these situations, it is necessary to differentiate urachal carcinoma from primary bladder adenocarcinoma and invasive adenocarcinoma originating from other sites. Specific histopathological and clinical criteria have been established to assist in this diagnosis.

### Histopathological Subtypes

Histological evaluation is the cornerstone of diagnosing UrC, with the most widely used criteria being those of Sheldon et al., Gopalan et al. and Mostofi et al., more recently, (3, 19, 59). These criteria encompass four main elements: (1) the tumor must be located in the bladder dome or anterior wall; (2) the tumor's epicenter must reside within the bladder wall; (3) there should be no evidence of extensive cystitis cystica or cystitis glandularis; and (4) the absence of a known primary adenocarcinoma in any other site. We found that the most frequent histological subtype was the mucinous adenocarcinoma of the urachus (40.1%), followed by enteric urachal adenocarcinoma (33.7%) and mixed adenocarcinoma of the urachus (5.8%).

### Tumoral staging

Tumor staging was reported using several classification systems. The Sheldon classification (59) was the most frequently used, with stage II being the most common (44%), followed by stage IIIa (23.6%) and stage IVa (8.5%). The Mayo classification (14) was available for 814 patients, where stage II was also the most prevalent (47.3%), followed by stage I (22%) and stage IV (19.3%). The TNM staging system was utilized in 579 patients, with stage T3 being the most common (54.5%), followed by stage T2 (25.5%) and stage IV (14.5%). Overall, only 15.6% of patients presented with metastatic disease at the time of primary diagnosis.

### Surgical treatment

The earliest historical series, including the two largest single-center studies by Begg in 1931 and Mostofi et al. in 1955, advocated for treatment with radical cystectomy combined with *en-bloc* resection of the urachus and umbilical region (2, 3, 58–60).

Currently, the standard treatment is primarily surgical, consisting of extended partial cystectomy with *en-bloc* resection of the urachal mass, urachal tract, and umbilicus, combined with pelvic lymph node dissection. Although radical cystectomy has been proposed as definitive therapy in some cases, it is generally reserved for larger tumors that involve more than the superior hemisphere of the bladder. Partial cystectomy is associated with fewer postoperative complications and improved quality of life (9, 15).

In our systematic review, surgical intervention was the primary treatment in 74.5% of cases, with partial cystectomy as the predominant approach for localized disease (80.8%), followed by radical cystectomy (11.5%). Open surgery was the most frequently reported surgical technique (21.8%), followed by laparoscopic (11.9%) and robotic-assisted approaches (3.1%). However, a significant portion of studies (63.1%) did not specify the surgical approach used. The treatment characteristics of the disease are detailed in Table-2.

**Table 2:** Disease management of urachal carcinoma.

Characteristic; n (%)		Overall population
**Primary treatment**		1,901
	Surgery	1,417 (74.54)
	Radiotherapy	37 (1.95)
	Chemotherapy	61 (3.21)
	Not specified	386 (20.31)
**Type of primary surgery**		1,417
	Partial cystectomy	1,145 (80.80)
	Radical cystectomy	163 (11.50)
	Transurethral Bladder Resection	50 (3.53)
	Not Specified	59 (4.16)
**Surgical technique**		1,828
	Open	349 (19.09)
	Laparoscopic	192 (10.50)
	Robotic	43 (2.35)
	Not specified	1,244 (68.05)
**Umbilectomy included**		948
	Yes	588 (62.0)
	No	360 (37.97)
**Lymphadenectomy**		1,640
	Yes	377 (22.98)
	No	444 (27.07)
	Not specified	819 (49.94)
**Extent of lymphadenectomy**		377
	Standard: obturator nodes, external iliac nodes, internal iliac nodes	167 (44.2)
	Extended: obturator nodes, external iliac nodes, internal iliac nodes, common iliac nodes, presacral nodes, and paravesical nodes.	17 (4.5)
	Not specified	193 (51.2)
**Lymph nodes at pathologic staging**		1147
	Positive	226 (19.7)
	Negative	921 (80.3)
**Number of lymph nodes removed; Mean (SD)**		10.26 (±3.99)

SD = Standard deviation

Sheldon et al. (3), after finding navel invasion in 7% of autopsies performed on patients who died because of urachal tumors, advocated surgical control of the urachal ligament via *en-bloc* excision of the bladder dome, urachal ligament, posterior rectus abdominis fascia, and umbilicus (15, 59, 61). However, some authors defend that the umbilectomy may be omitted in patients with localized lesions to avoid impact on body image and quality of life (37, 62). In our systematic review, we identified 360 patients (38%) who did not undergo umbilical resection across 14 studies that reported not performing umbilectomy with urachal *en-blo*c excision (14, 15, 17, 18, 20–22, 26–28, 31, 37, 40, 44). Of the five studies comparing patients who underwent umbilectomy to those who did not, four reported worse overall survival, cancer-specific survival, and progression-free survival in patients who did not receive complete urachal remnant resection with umbilectomy. Although one study found no statistically significant difference in survival (p=0.09), the Kaplan-Meier curve suggested a trend, with 13 of the 16 long-term survivors in the group that underwent *en-bloc* resection with umbilectomy, as shown in Table-3.

**Table 3:** Comparison of survival outcomes between patients who underwent umbilectomy and those who did not.

Author	Year of publication	N0 of patients underwent umbilectomy	N0 of patients not underwent umbilectomy	Survival data
**Yu, et al. (** **45** **)**	2021	12	191	Overall survival: HR 2.491; 95% 0.980 - 6.334; p=0.005 Cancer-Specific Survival: HR 2.601; 95%CI 1.024 - 6.608; p=0.044 Recurrence-free survival: HR 2.140; 95%CI 0.918 - 4.990; p=0.078
**Ashley, et al. (** **15** **)**	2006	32	27	Cancer specific survival: HR 3.0; 95%CI 1.3 - 6.8; p=0.008
**Siefker-Radtke, et al. (** **32** **)**	2016	19	16	*En-bloc* resection was not statistically associated with survival (p = 0.09), but 13 of the 16 long-term survivors after resection were in the group treated with *en-bloc* resection and umbilectomy.
**Jia, et al. (** **41** **)**	2020	27	12	Overall survival in the umbilectomy group: HR = 0.141; 95% CI = 0.034–0.591, p=0.007. Progression-free survival: HR = 0.355; 95% CI = 0.128–0.983, p=0.046. Patients who underwent umbilectomy had significantly longer median overall survival (87 vs. 48 months, p=0.03) and progression-free survival (67 vs. 31 months, P=0.036) than those who did not.
**Dhillon, et al. (** **29** **)**	2015	29	11	Patients underwent umbilectomy: 10 died of cancer (34%) in a mean of 35 months (range, 13-74 months). Patients who did not undergo umbilectomy: 7 died of cancer (64%) in a mean of 31 months (range, 12-71 months).

HR = Hazard Ratio; 95%CI = 95% Confidence interval

Before this review, the conduct of umbilectomy with *en-bloc* resection of the urachal tract was based on the earlier study of Sheldon et al. (3). This systematic review presents five studies from the literature that highlight the oncological benefits of umbilectomy with *en-bloc* resection, further reinforcing this approach as the standard treatment for patients with localized UrC.

Concerning the role of lymphadenectomy, 13 studies (14, 16–18, 21, 22,31, 37, 40, 41, 44, 49, 50) reported patients who did not undergo pelvic lymphadenectomy within the overall cohort. Still, only 3 studies compared the oncological outcomes between the two groups. Duan et al. reported that among the 35 patients who did not undergo lymphadenectomy, 7 (20%) experienced nodal recurrence, whereas 3 (11.1%) of the 27 patients who underwent lymphadenectomy had nodal recurrence (42). However, in the authors’ analysis, performing pelvic lymphadenectomy was not correlated with disease-free survival (42). The second study comparing both groups included 20 patients who underwent lymphadenectomy and 40 patients who did not. The authors reported that lymphadenectomy predicted cancer-specific mortality in the univariate analysis (p = 0.02; HR 1.5, 95% CI 0.7–2.8) (14). Lastly, a third article reported 18 patients who underwent lymphadenectomy and 16 who did not. After performing a survival analysis, the authors found that lymphadenectomy had no positive effect on survival (40). The evidence in the literature remains limited, as most series have not evaluated the association between pelvic lymphadenectomy and oncological outcomes. Among the few studies that do address this, there is no clear specification regarding the extent of lymphadenectomy performed, and the results are often conflicting (63).

### Systemic treatment

The NCCN recommends chemotherapy regimens for node-positive bladder adenocarcinoma that are similar to those used in colorectal cancer treatment. Specifically, the FOLFOX regimen (oxaliplatin, leucovorin, and 5-fluorouracil) and the GemFLP regimen (5-fluorouracil, leucovorin, gemcitabine, and cisplatin) are suggested as potential options (62).

For advanced disease, participation in clinical trials is strongly recommended. Although, in cases where trial enrollment is not feasible, combination chemotherapy may be an option with regimens based on 5-Fluorouracil (FOLFOX or GemFLP) or with ITP (paclitaxel, ifosfamide and cisplatin) or dual therapy with paclitaxel and a platinum compound (62–65).

**Figure 2:**
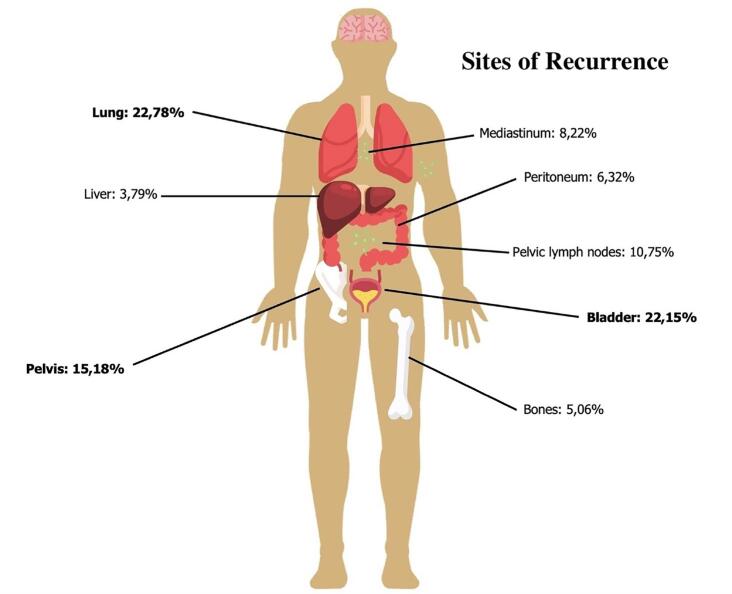
Main sites of tumor recurrence in patients with urachal carcinoma following primary treatment.

In this systematic review, 16.2% of patients presented with metastatic disease at the initial diagnosis, and 16% experienced tumor recurrence after primary treatment. The primary site of tumor recurrence was the lung (22.8%), followed by the bladder (22.1%) and the pelvis (15.2%), as shown in Figure-2.

In the studies with oncological outcomes, neoadjuvant therapy was administered to only 8 patients; however, chemotherapy regimens and response data were not available for 3 of these patients. Among the remaining patients receiving neoadjuvant therapy, treatment and outcomes were as follows: 4 cycles of gemcitabine and cisplatin were administered, though response data were unavailable; a 5-fluorouracil and cisplatin regimen achieved a partial response, with the patient remaining disease-free at study conclusion (median follow-up post-surgery was 17 months); and a regimen combining ifosfamide, docetaxel, and cisplatin was associated with disease progression.

A total of 190 patients (9.9%) received adjuvant therapy, with specific chemotherapy regimens reported for 72% of cases, covering 32 unique regimens. Cisplatin was included in 51% of adjuvant regimens, while 5-fluorouracil was used in 26%. Among patients with recurrence or metastatic disease, 93 (4.9%) received systemic treatment, encompassing 24 distinct regimens; cisplatin was administered in 49.5% of these cases, while 5-fluorouracil was included in 46.2%.

Therapeutic responses were documented for 112 patients, 65 patients (58%) who received adjuvant therapy, and 47 patients (42%) who received systemic salvage treatment. In the adjuvant cohort, 63% presented no disease progression, whereas in the metastatic or recurrent group, 14.9% showed a partial or complete response.

In adjuvant therapy, patients treated with a 5-fluorouracil-based regimen showed a 60% rate of no disease progression. Among those receiving cisplatin-based therapy, 65.73% remained disease-free. None of the three patients who received a combination of 5-fluorouracil and cisplatin experienced disease progression.

In salvage treatment, 15.4% of those treated with a cisplatin-based regimen achieved a complete or partial response, while 14.8% of patients on a combined regimen of 5-fluorouracil and cisplatin showed complete or partial response. Only one patient who received a 5-fluorouracil-based regimen had documented oncological outcomes and demonstrated a complete or partial response. In terms of disease stability in the metastatic or recurrent setting, 30.7% of patients treated with a cisplatin-based regimen maintained stable disease, whereas 37% of those on combined 5-fluorouracil and cisplatin regimens achieved disease stability. Supplementary Table-2 lists all systemic treatments used.

## ONCOLOGICAL OUTCOMES

**Figure 3:**
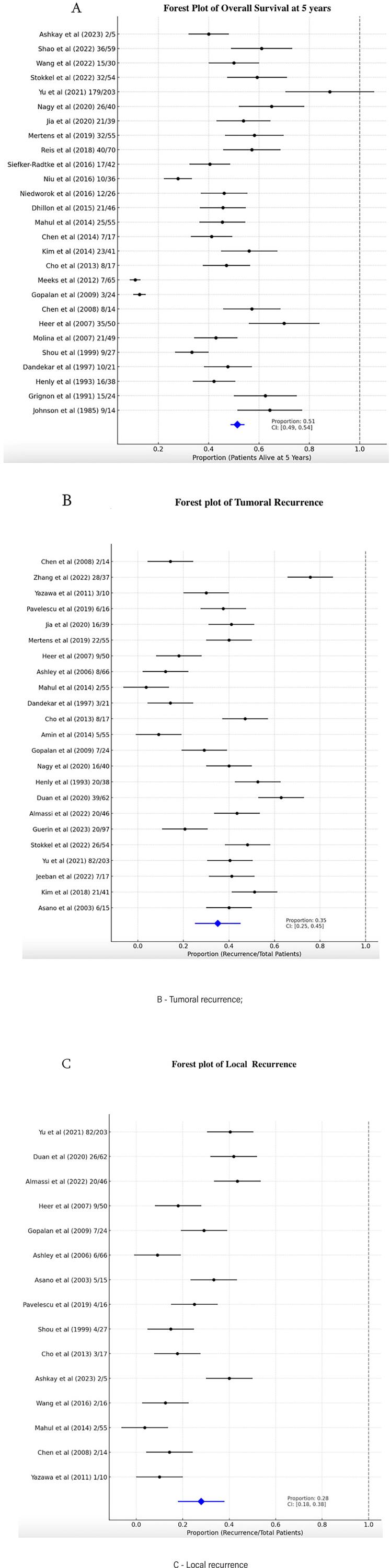
Forest plot of oncological outcomes.

The single-arm meta-analysis indicated a 5-year overall survival rate of 51% (95% CI 0.49–0.54). Tumor recurrence was observed in 35% of cases (95% CI 0.25–0.45), with local recurrence occurring in 28% of cases (95% CI 0.18–0.38). The mean time to recurrence was 27.6 months. Figure 3 presents a forest plot illustrating the oncological outcomes.

## CONCLUSIONS

Urachal carcinoma is a rare malignancy with limited and heterogeneous evidence guiding its management. This meta-analysis, encompassing the largest patient cohort to date, provides a more robust foundation for clinical decision-making in this uncommon disease. Our findings emphasize the oncological benefits of *en-bloc* resection with umbilectomy for affected patients. In the adjuvant setting, regimens containing 5-fluorouracil and cisplatin demonstrated the most efficacy, while cisplatin-based chemotherapy showed favorable responses in metastatic cases. Furthermore, the response rates observed with alternative regimens suggest a potential role for emerging systemic therapies in the treatment of urachal carcinoma.

## APPENDIX

**Supplementary Table 1:** Studies included in the analysis.

Author	Article Year	Country
Johnson, et al. (8)	1985	USA
Grignon, et al. (9)	1991	USA
Henly, et al. (10)	1993	USA
Dandekar, et al. (11)	1997	India
Shou, et al. (12)	1999	China
Asano, et al. (13)	2003	Japan
Thali-Schwab, et al. (14)	2005	USA
Ashley, et al (15)	2006	USA
Heer, et al (16)	2007	USA
Molina, et al (17)	2007	USA
Chen, et al (18)	2008	China
Gopalan, et al (19)	2009	USA
Paner, et al (20)	2011	USA
Yazawa, et al (21)	2011	Japan
Meeks, et al (22)	2012	USA
Cho, et al (23)	2013	South Korea
Kim, et al (24)	2014	South Korea
Ke, et al (245	2023	China
Jung, et al (26)	2014	South Korea
Chen, et al (27)	2014	China
Amin, et al (28)	2014	USA
Dhillon, et al (29)	2015	USA
Wang, et al (30)	2016	China
Niu, et al (31)	2016	China
Siefker-Radtke, et al (32)	2003	USA
Niedworok, et al (33)	2016	Germany
Xing Bi, et al (34)	2017	China
Hang, et al (35)	2017	China
Reis, et al (36)	2018	Germany
Kim, et al (37)	2018	South Korea
Pavelescu, et al (38)	2019	Romania
Mertens, et al (39)	2019	United Kingdom
Cornejo, et al (40)	2020	USA
Jia, et al (41)	2020	China
Duan, et al (42)	2020	China
Nagy, et al (43)	2020	Hungary
P Das, et al (44)	2022	USA
Yu, et al (45)	2021	South Korea
Almassi, et al (46)	2022	USA
Wang, et al (47)	2022	China
Jeeban, et al (48)	2022	USA
Zhang, et al (49)	2022	China
Shao, et al (50)	2022	China
Stokkel, et al (51)	2022	Netherlands
Stokkel, et al (52)	2023	Netherlands
Varadi, et al (53)	2023	Hungary
Guerin, et al (54)	2023	France
Ashkay, et al (55)	2023	USA
Sang, et al (56)	2023	South Korea
Suartz, et al (57)	2024	Brazil

**Supplementary Table 2:** Systematic treatment.

Characteristic		Overall population
Neoadjuvant therapy received; n (%)		8 (0.42)
**Type of neoadjuvant treatment; n (%)**		
	Gemcitabine and Cisplatine	2 (25)
	5-fluorouracil and cisplatin-based regimen	2 (25)
	Ifosfamide, docetaxel, and cisplatin	1 (12.5)
	Not specified	3 (37.5)
Adjuvant therapy received; n (%)		190 (9.99)
**Type of adjuvant therapy; n (%)**		
	Cisplatin and Paclitaxel	5 (2.63)
	5-Fluorouracil, doxorubicin, mitomycin	2 (1.05)
	Cisplatin-based	19 (10.00)
	Paclitaxel	3 (1.58)
	Doxorubicin	2 (1.05)
	5-Fluorouracil based	3 (1.58)
	MVAC (methotrexate, vinblastine, doxorubicin, cisplatin)	4 (2.11)
	Taxol and platinum	3 (1.58)
	Etoposideo and platinum	1 (0.53)
	Gencitabine and cisplatin	12 (6.32)
	5-Fluorouracil and cisplatin/carboplatin	20 (10.53)
	5-Fluorouracil, cisplatin and gencitabine	7 (3.68)
	5-Fluorouracil, cisplatin and doxorrubicine	6 (3.16)
	5-Fluorouracil, doxorubicin and etoposide	2 (1.05)
	Cisplatin and Nivolumab	2 (1.05)
	Bevacizumab and unspecified chemotherapy	2 (1.05)
	Gemcitabine, Cisplatin, Afatinib, Tegafur, Gimeracil, Oteracil and Paclitaxel	1 (0.53)
	5-fluorouracil or gemcitabine and/or cisplatin.	11 (5.79)
	Gemcitabine or Capecitabine combined with Cisplatin or Oxaliplatin	11 (5.79)
	Capecitabine combined with Taxol	3 (1.58)
	Capecitabine combined with Gemcitabine	2 (1.05)
	Taxol combined with Cisplatin or 5-fluorouracil	2 (1.05)
	Pembrolizumab	2 (1.05)
	Folinic acid, 5-fluorouracil, and oxaliplatin	3 (1.58)
	Carboplatin	1 (0.53)
	Neratinib	1 (0.53)
	Atezolizumab	1 (0.53)
	Ipilimumab	1 (0.53)
	Nivolumab	1 (0.53)
	Capecitabina and oxaliplatin	1 (0.53)
	Cisplatin and paclitaxel	1 (0.53)
	Capecitabine	1 (0.53)
	Paclitaxel, ifosfamide and cisplatin	1 (0.53)
	Not specified	53 (27.89)
Systemic salvage treatment received; n (%)		93 (4.89)
**Type of Salvage Chemotherapy; n (%)**		
	5-Fluorouracil and irinotecan	1 (1.08)
	5-Fluorouracil or gemcitabine and/or cisplatin.	8 (8.60)
	5-Fluorouracil, doxorubicin, and cisplatin	1 (1.08)
	5-Fluorouracil, doxorubicin, mitomycin	3 (3.23)
	5-Fluorouracil, leucovorin, and oxaliplatin	1 (1.08)
	5-Fluorouracil, leucovorin, gemcitabine and cisplatin	6 (6.45)
	5-Fluorouracil, mitomycin C, and mitoxantrone	1 (1.08)
	5-Fluorouracil, mitomycin, cisplatin and doxorubicin	4 (4.30)
	5-Fluorouracil, α-interferon, cisplatin	3 (3.23)
	5-Fluouracil and cisplatin	1 (1.08)
	5-Fluouracil based	14 (15.05)
	Cisplatin ifosfamide and gemcitabine	1 (1.08)
	Cisplatin-based	7 (7.53)
	Cisplatin, gemcitabine and etoposide	1 (1.08)
	Cyclophospamide	3 (3.23)
	Docetaxel and cisplatin	1 (1.08)
	Doxorubicin	5 (5.38)
	Doxorubicin, cisplatin, and mitomycin C	1 (1.08)
	Gemcitabine, cisplatin	1 (1.08)
	Methotrexate, vinblastine, doxorubicin, cisplatin	5 (5.38)
	Mytomycin, cisplatin, cyclophosphamide	1 (1.08)
	Paclitaxel	3 (3.23)
	Paclitaxel and carboplatin	2 (2.15)
	Paclitaxel and cisplatin	2 (2.15)
	Paclitaxel, methotrexate and cisplatin	1 (1.08)
	Not specified	16 (17.20)
